# Efficacy and safety of blinatumomab in Chinese patients with relapsed/refractory B-cell acute lymphoblastic leukemia: a single-center retrospective study

**DOI:** 10.3389/fonc.2025.1587185

**Published:** 2025-05-12

**Authors:** Yan Wang, Chun Chang, Zhen Wang, Jie Zhao, Jingbo Wang

**Affiliations:** Department of Hematology, Aerospace Center Hospital, Beijing, China

**Keywords:** acute lymphoblastic leukemia, relapsed/refractory, blinatumomab, complete remission, minimal residual disease

## Abstract

**Background:**

Blinatumomab is a bispecific T-cell engager approved for the treatment of relapse/refractory B-cell acute lymphoblastic leukemia (R/R B-ALL). Most studies evaluating blinatumomab were conducted in Caucasian populations, with limited data available in Chinese patients. This retrospective study aims to evaluate the efficacy and safety of blinatumomab in Chinese patients with R/R B-ALL.

**Methods:**

A total of 19 patients (10 males, 9 females) with a median age of 49 years (range: 9-66) who received blinatumomab treatment at the Aerospace Center Hospital between November 2021 and November 2024 were included. Rates of complete remission (CR) and minimal residual disease (MRD) response, 1-year overall survival (OS) and relapse-free survival (RFS), and adverse events were analyzed.

**Results:**

The median number of blinatumomab cycles administered was 1 (range: 1-6). Twelve (80.0%, 95% confidence interval [CI]: 51.9-95.7) of the 15 patients with overt marrow disease achieved CR, with 8 achieving MRD negativity. Four patients with < 5% blast but positive MRD all sustained CR and achieved MRD negativity. The overall MRD response rate was 63.2% (12/19, 95%CI: 38.4-83.7). The 1-year overall survival (OS) and relapse-free survival (RFS) rates were 64.2% ± 12.1% and 73.3% ± 11.4%, respectively. MRD responders had significantly better OS compared to MRD non-responders (log-rank test, P = 0.023). Of the 16 patients with CR, 62.5% proceeded to allogeneic hematopoietic stem cell transplantation (allo-HSCT). The most frequent adverse event was cytokine release syndrome, which occurred in 11 patients (10 with grade 1–2 and 1 with grade 3 severity).

**Conclusion:**

Blinatumomab is both effective and well-tolerated in Chinese patients with R/R B-ALL, achieving high rates of CR and MRD negativity and facilitating more patients’ eligibility for allo-HSCT.

## Introduction

B-cell acute lymphoblastic leukemia (B-ALL) is the most common type of ALL, accounting for 70% of all cases (1–3). Most newly-diagnosed B-ALL patients achieve complete remission (CR) following induction chemotherapy (4). In pediatric patients, the 5-year overall survival (OS) rate approaches 90%, and the 10-year OS rate exceeds 85% (5). However, 15% to 20% of pediatric patients experience relapse or refractory disease (6). In contrast, adult patients have a significantly lower long-term survival rate (20%-40%), with more than half eventually relapsing or becoming refractory to chemotherapy (7, 8). Only 20% to 30% of relapse/refractory (R/R) B-ALL patients achieve CR after reinduction by conventional chemotherapy (9), resulting in a 5-year OS rate as low as 10% (10). Allogeneic hematopoietic stem cell transplantation (allo-HSCT) is a curative option for R/R B-ALL; however, only a small proportion of these patients are able to undergo this procedure due to failure to achieve CR after salvage chemotherapy or due to chemotherapy-related toxicities, which make them unsuitable for transplantation (11). Therefore, novel treatment strategies with high response rates and minimal toxicity will allow more R/R B-ALL patients to proceed to transplantation and improve long-term survival.

Blinatumomab, a bispecific T-cell engaging (BiTE) antibody, targets CD19+ B cells and CD3+ T cells, redirecting cytotoxic T cells to recognize and eliminate CD19+ B cells, including those in B-ALL (12, 13). A phase II, single-arm trial demonstrated that 43% of Philadelphia chromosome-negative (Ph-) R/R ALL patients treated with blinatumomab achieved a CR or CR with partial hematologic recovery (CRh) (14). A phase III, randomized controlled trial further showed that blinatumomab significantly improved CR rate (34% *vs.* 16%) and prolonged OS (median 7.7 months *vs.* 4.0 months) compared to chemotherapy (15). Additionally, 78% of B-ALL patients with minimal residual disease (MRD) positivity achieved a complete MRD response following blinatumomab treatment (16). Based on the results from clinical trials demonstrating its efficacy and safety (14–19), the Food and Drug Administration (FDA) approved blinatumomab for the treatment of Ph- R/R B-ALL in 2014, Ph+ R/R B-ALL in 2017, and MRD-positive ALL in 2018 (20).

Most studies evaluating blinatumomab have been conducted in Caucasian populations, with limited data available on its efficacy and safety in Chinese patients. This single-center, retrospective study aims to evaluate the efficacy and safety of blinatumomab in Chinese patients with R/R B-ALL.

## Materials and methods

### Participants

This single-center study retrospectively collected data from patients with R/R B-ALL who received blinatumomab treatment at the Department of Hematology, Aerospace Center Hospital, China between November 2021 and November 2024. B-ALL was diagnosed according to the 2016 version of the World Health Organization Classification of Tumors of Hematopoietic and Lymphoid Tissues (21). Refractory B-ALL patients was defined by any of the following criteria: (1) failure to achieve CR after induction therapy; (2) relapse within 6 months after CR1; (3) relapse after 6 months of CR2 but failure to achieve CR following subsequent induction therapy; (4) two or more relapses; (5) extramedullary leukemia (22, 23). Relapse B-ALL was defined as reappearance of leukemic cells in peripheral blood, ≥ 5% blast in bone marrow aspirates, and/or extramedullary disease after documented CR (22, 23). Only patients who had completed at least one cycle of blinatumomab and underwent bone marrow assessment were included in the analysis. This study was conducted in accordance with the Declaration of Helsinki and approved by the Ethics Committee of Aerospace Center Hospital. Written informed consent was obtained from all patients or their guardians.

### Blinatumomab treatment and data collection

For patients with a body weight ≥ 45 kg, blinatumomab was administered at a dose of 9 μg/day from days 1 to 7 and 28 μg/day from days 8 to 14 or 28. For patients weighting < 45 kg, the dose was adjusted according to body surface area (5 μg/m^2^/day from days 1 to 7, 15 μg/m^2^/day from days 8 to 14 or 21). Bone marrow assessments were conducted on day 8 of blinatumomab infusion and within two weeks after treatment. Demographic information, clinical, and laboratory data, as well as details of treatment and transplantation, were collected from the electronic medical records. Follow-up data were acquired through telephone contact and during each hospital visit for treatment.

### Outcomes

The primary outcomes were the rates of CR and minimal residual disease (MRD) response. MRD response was defined as the conversion from MRD positivity (≥ 0.01%) to MRD negativity (< 0.01%) following blinatumomab treatment, as detected by multiparameter flow cytometry (MFC) with a sensitivity of 0.01% in bone marrow aspirates. Secondary outcomes included OS, RFS, and adverse events (AEs). OS was defined as the time interval from the start of blinatumomab infusion to death from any cause or the last follow-up. RFS was calculated from the initiation of blinatumomab to the date of relapse, death from any cause, or the last follow-up. AEs were graded according to the Common Terminology Criteria for Adverse Events (CTCAE) Version 5.0, and cytokine release syndrome (CRS) was graded based on the American Society for Transplantation and Cellular Therapy Consensus Grading (24).

### Statistical analysis

Categorical variables are presented as counts and percentages and were compared using the chi-square test or Fisher’s exact test. Continuous variables are presented as medians with ranges. OS and RFS were estimated using the Kaplan-Meier method and compared by the log-rank test. A p-value of < 0.05 was considered statistically significant. All statistical analyses were performed using STATA v16 (StataCorp, TX, USA).

## Results

### Patient characteristics

A total of 19 R/R B-ALL patients (10 males, 9 females) were included in the analysis (**Table 1**). The median age at the time of blinatumomab treatment was 49 years (range: 9-66). The median time from diagnosis to blinatumomab treatment was 12 months (range: 2-84). Nine patients were diagnosed with Philadelphia-positive (Ph+) B-ALL. Four patients had previously received allogeneic hematopoietic stem cell transplantation (allo-HSCT) and two had received chimeric antigen receptor T-cell therapy (CAR-T) before blinatumomab. At the start of blinatumomab infusion, four patients had < 5% bone marrow blasts, while 15 patients had overt marrow disease with ≥ 5% blast. All patients were MRD positive (≥ 0.01%). The median number of blinatumomab cycles was 1 (range: 1-6). Twelve patients (63.2%) received one cycle of blinatumomab infusion, and four (21.1%) underwent two cycles. Two patients underwent one cycle of blinatumomab re-induction followed by five cycles of consolidation.

**Table 1:** Baseline characteristics of R/R B-ALL patients.

Characteristics	n = 19
Sex, n (%)	
Male	10 (52.6)
Female	9 (47.4)
Age, median (range), years	49 (9-66)
≤ 18	4 (21.1)
> 18	15 (78.9)
BM blast % on staring blinatumomab, median (range)	26 (0-96)
BM blast category, n (%)	
< 5%	4 (21.1)
5%-49%	8 (42.1)
≥ 50%	7 (36.8)
MRD status on starting blinatumomab, n (%)	
Positive (≥ 0.01%)	19 (100)
Philadelphia chromosome, n (%)	
Ph+	9 (47.4)
Ph-	10 (52.6)
Months from diagnosis to blinatumomab infusion, median (range)	12 (2-84)
Prior allo-HSCT, n (%)	4 (21.1)
Prior CAR-T, n (%)	2 (10.5)
EMD before blinatumomab treatment, n (%)	6 (31.6)
CNS involvement before blinatumomab treatment, n (%)	2 (10.5)
Blinatumomab courses, n (%)	
1	12 (63.2)
2	5 (26.3)
> 2	2 (10.5)
Allo-HSCT after blinatumomab treatment, n (%)	11 (57.9)
HID	7 (63.6)
MSD	2 (18.2)
MUD	2 (18.2)
Days between blinatumomab and HSCT, median (range)	26 (7-78)

Allo-HSCT, allogeneic hematopoietic stem cell transplantation; CAR-T, chimeric antigen receptor T-cell therapy; CNS, central nervous system; EMD, extramedullary disease; HID, haploidentical donor; MRD, minimal residual disease; MSD, matched sibling donor; MUD, matched unrelated donor; R/R B-ALL, relapsed/refractory B-cell precursor acute lymphoblastic leukemia.

### Adverse events

Twelve patients (63.2%) experienced at least one AEs during blinatumomab infusion (**Table 2**). The most common AEs was CRS, which occurred in 11 patients. Most cases of CRS were mild (grade 1-2), with only one patient experienced grade 3 CRS. Three patients developed grade 3 febrile neutropenia, and two experienced bacterial sepsis. Grade 3 cardiovascular events occurred in two patients (one with heart failure and one with cardiac insufficiency). One patient experienced a grade 1 headache. One patient temporarily interrupted treatment due to hepatotoxicity (grade 4 elevated liver enzymes), while another had treatment interruption due to grade 3 CRS; both resumed treatment after appropriate interventions. In one patient, the dose of blinatumomab was reduced from 28 μg/day (days 8-10) to 14 μg/day (days 11-28) due to AEs (grade 4 edema, grade 3 cardiac insufficiency). One patient discontinued treatment after 14 days of infusion due to multiple AEs, including febrile neutropenia, bacterial sepsis and heart failure.

**Table 2:** AEs during blinatumomab infusion.

AEs	Grade 1-2, n (%)	Grade ≥ 3, n (%)	Total, n (%)
Patients with at least one AEs	–	–	12 (63.2)
Febrile neutropenia	0	3 (15.8)	3 (15.8)
CRS	10 (52.6)	1 (5.3)	11 (57.9)
Elevated liver enzyme	0	1 (5.3)	1 (5.3)
Nausea	1 (5.3)	0	1 (5.3)
Vomiting	1 (5.3)	0	1 (5.3)
Bacterial sepsis	0	2 (10.5)	2 (10.5)
Edema	0	1 (5.3)	1 (5.3)
Fatigue	1 (5.3)	0	1 (5.3)
Cardiovascular event	0	2 (10.5)	2 (10.5)
Headache	1 (5.3)	0	1 (5.3)

AEs, adverse events; CRS, cytokine release syndrome.

### Treatment response and post-blinatumomab treatments

Among the four patients with < 5% blast and MRD positivity at the start of blinatumomab, all sustained CR and achieved MRD negativity after 1–2 cycles of treatment. Twelve (80.0%) of the 15 patients with ≥ 5% blast at the start achieved CR, with 8 achieving MRD negativity. The other three patients did not response to blinatumomab treatment. Therefore, the CR rate among patients with > 5% blast was 80.0% (12/15, 95% confidence interval [CI]: 51.9-95.7), and the MRD response rate of all patients was 63.2% (12/19, 95%CI: 38.4-83.7). As shown in **Table 3**, the rates of CR and MRD response did not significantly differ across patient subgroups based on sex (male *vs.* female), age (≤ 18 *vs.* > 18 years), BM blast percentage (< 50% *vs.* ≥ 50%), Philadelphia chromosome status (Ph+ *vs.* Ph-), time from diagnosis to treatment (< 12 months *vs.* ≥ 12 months), or the presence of extramedullary disease before treatment (yes *vs.* no). Among patients with overt marrow disease, two had received allo-HST previously but both did not obtain CR after blinatumomab treatment, whereas 12 of 13 patients without previous transplantation achieved CR after blinatumomab (Fisher’s exact test, P = 0.029). MRD response did not significantly differ between patients with or without previous transplantation.

**Table 3:** Treatment response after blinatumomab treatment.

Subgroups	CR, n/total (%)	MRD response, n/total (%)
All patients	12/15 (80.0)	12/19 (63.2)
Sex		
Male	6/8 (75.0)	6/10 (60.0)
Female	6/7 (85.7)	6/9 (66.7)
Age, years		
≤ 18	3/4 (75.0)	3/4 (75.0)
> 18	9/11 (81.2)	9/15 (60.0)
BM blast before blinatumomab		
< 50%	6/8 (75.0)	8/12 (66.7)
≥ 50%	6/7 (85.7)	4/7 (57.1)
Philadelphia chromosome		
Ph+	5/6 (83.3)	5/9 (55.6)
Ph-	7/9 (77.8)	7/10 (70.0)
Months from diagnosis to treatment		
< 12	5/6 (83.3)	8/9 (88.9)
≥ 12	7/9 (77.8)	4/10 (40.0)
EMD before blinatumomab		
Yes	5/6 (83.3)	3/6 (50.0)
No	7/9 (77.8)	9/13 (69.2)
Previous allo-HSCT		
Yes	0/2 (0)	2/4 (50.0)
No	12/13 (92.3)^#^	10/15 (63.3)

^#^Fisher’s exact test P = 0.029.

CR, complete remission.

A total of 11 patients underwent allo-HSCT during the follow-up period. The median time from blinatumomab treatment to HSCT was 26 days (range: 7-78). Of these, 9 patients with CR (7 MRD responders and 2 MRD non-responders) proceeded directly to HSCT without additional bridging therapy. One patient who was MRD non-responsive received imatinib prior to HSCT. All of these three MRD non-responders achieved MRD negativity post-transplant. One patient without MRD response who experienced early bone marrow relapse after one cycle of blinatumomab received azacitidine, ponatinib, and a CD22 monoclonal antibody before HSCT and subsequently achieved CR with MRD negativity. Two patients who did not achieve CR after blinatumomab treatment received further therapies followed by CAR-T.

### Survival outcomes

At a median follow-up of 11.0 months (range: 1.7-28.5), 12 patients (63.2%) were still alive, all in CR with MRD negativity. Seven patients (36.8%) died and three patients (15.8%) experienced relapse during the follow-up period. Of the three patients who did not achieve CR after blinatumomab, two died from disease progression, and one died from severe infection during post-blinatumomab CAR-T. Another patient, who achieved CR and received allo-HSCT, also died from severe infection. One patient who experienced bone marrow and central marrow system relapse after one cycle of treatment received a second cycle of blinatumomab but eventually died from disease progression. Another patient, who had bone marrow relapse after allo-HSCT, achieved CR with MRD negativity after a second cycle of blinatumomab but later died from chronic graft-versus-host disease. One patient who had bone marrow relapse achieved CR again after multiple therapies and allo-HSCT, but later died from heart failure and renal failure.

**Figure 1:**
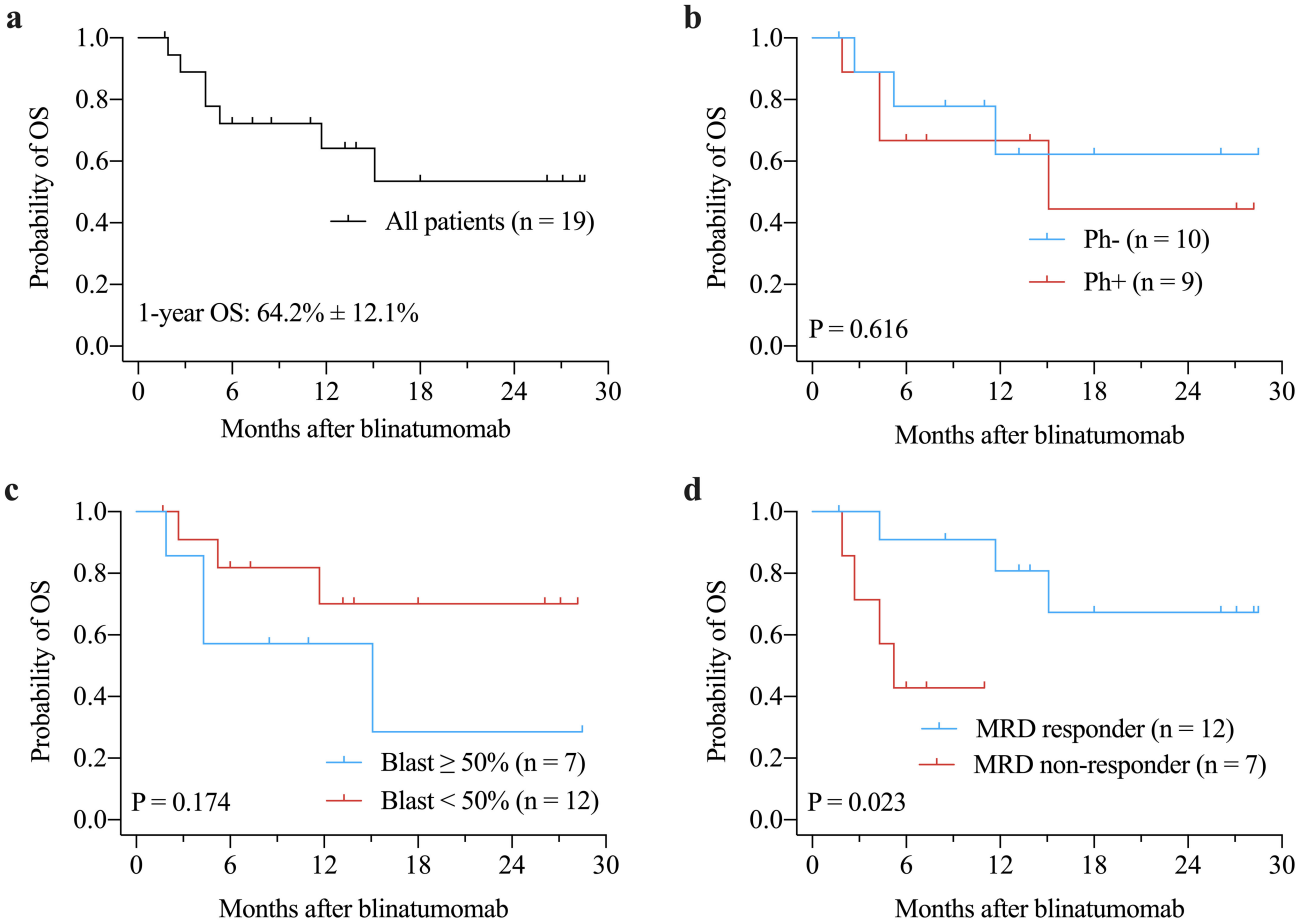
Kaplan-Meier curves of OS for the entire cohort **(a)**, and for subgroups according to Philadelphia chromosome status **(b)**, bone marrow blast prior to blinatumomab treatment **(c)**, and MRD response **(d)**. MRD, minimal residual disease; OS, overall survival.

**Figure 2:**
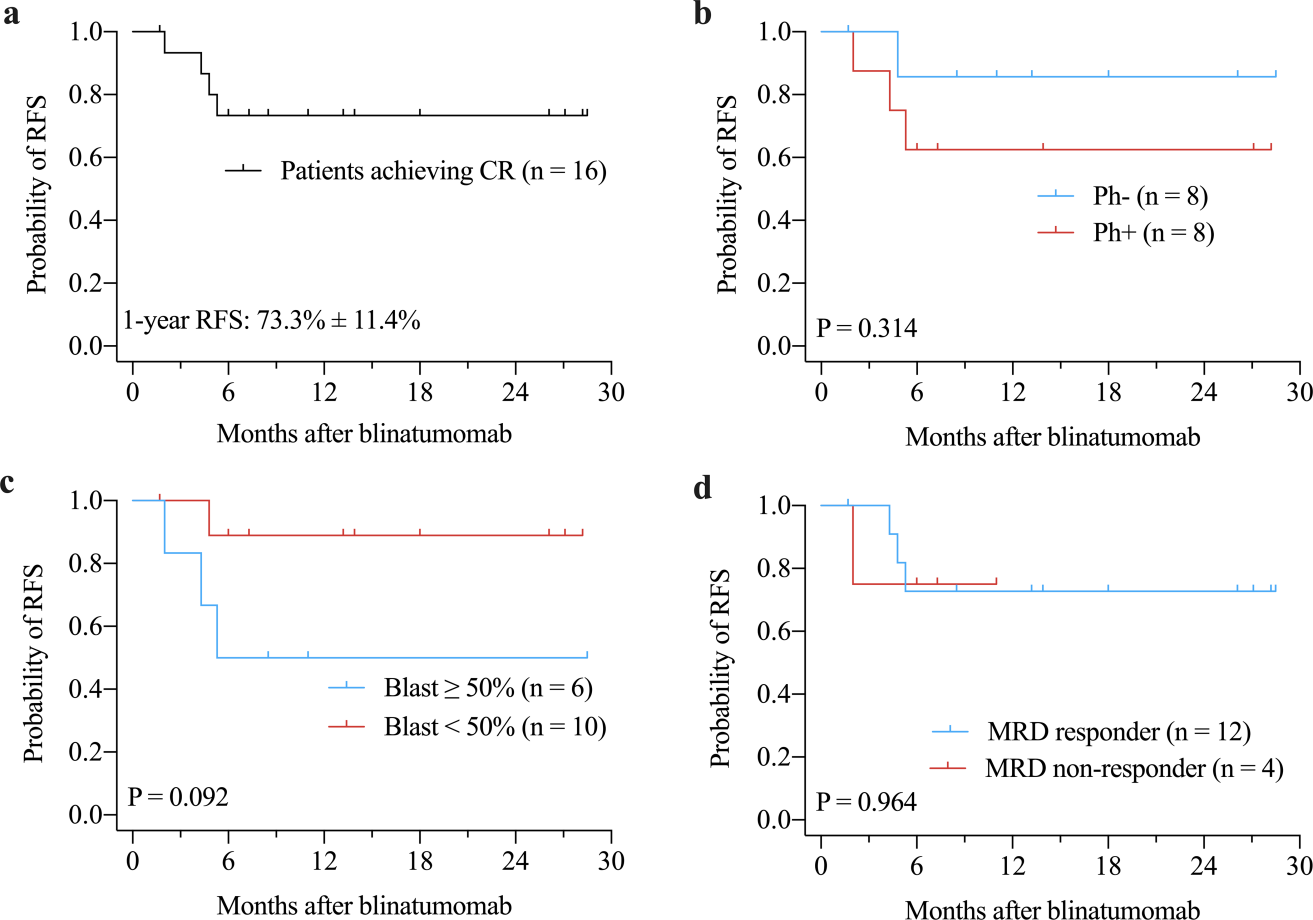
Kaplan-Meier curves of RFS for patients achieving CR **(a)**, and for subgroups according to Philadelphia chromosome status **(b)**, bone marrow blast prior to blinatumomab treatment **(c)**, and MRD response **(d)**. CR, complete remission; MRD, minimal residual disease; RFS, relapse-free survival.

**Figure 3:**
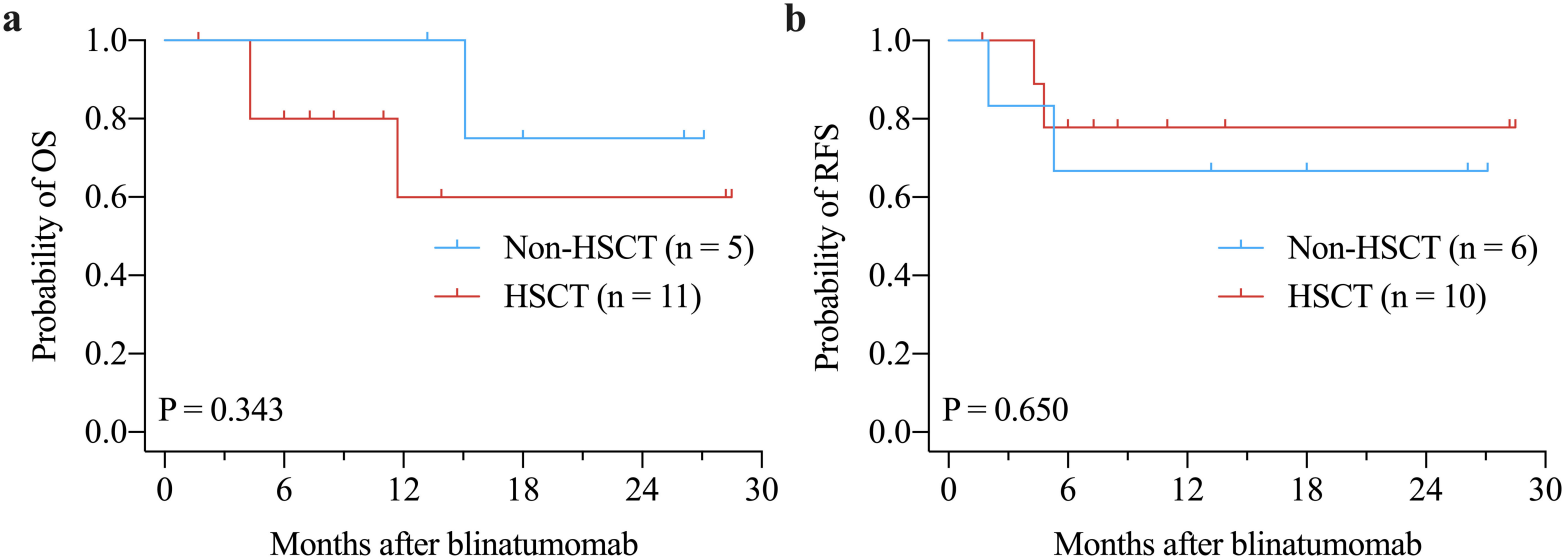
Kaplan-Meier curves of OS **(a)** and RFS **(b)** in patients achieving CR by allo-HSCT after blinatumomab treatment. allo-HSCT: allogeneic hematopoietic stem cell transplantation; CR: complete remission; OS: overall survival; RFS: relapse-free survival.

The median OS and RFS were not reached. The 1-years OS rate for the entire cohort was 64.2% ± 12.1% (**Figure 1a**). There were no significant differences in OS between Ph+ and Ph- patients (P = 0.616, **Figure 1b**) or between patients with ≥ 50% blast *vs.* < 50% blast before blinatumomab treatment (P = 0.174, **Figure 1c**). However, MRD responders had significantly better OS compared to MRD non-responders (P = 0.023, **Figure 1d**). The 1-year RFS rate for patients with CR was 73.3% ± 11.4% (**Figure 2a**). No significant differences in RFS were observed between in Ph+ *vs.* Ph-, ≥ 50% blast *vs.* < 50% blast, or MRD responders *vs.* non-responders (**Figures 2b–d**). Among the 16 patients obtaining CR, there were no significant differences in OS (**Figure 3a**) or RFS (**Figure 3b**) between patients who did or did not undergo allo-HSCT.

## Discussion

Blinatumomab is the first CD19/CD3 bispecific T cell-engaging monoclonal antibody approved for the treatment of adult and pediatric patients with R/R B-ALL and MRD (25). CD19 is a surface marker of B lymphocytes and is widely expressed in leukemia and lymphoma derived from the B-cell lineage, while CD3 is a critical marker expressed on the surface of cytotoxic T lymphocytes (26). Blinatumomab facilitates the interaction between CD3 on T cells and CD19 on both benign and malignant B cells, promoting the transient cytolytic synapse formation between T cells and tumor cells (27). This process upregulates cell adhesion molecules, increases the production of proteolytic enzymes, enhances the release of inflammatory cytokines, and promotes T-cell proliferation, ultimately leading to the lysis of CD19+ cells (28, 29). Notably, normal cells lacking CD19 expression are not affected by CD19-targeted therapy (29). Due to this unique mechanism, blinatumomab has demonstrated highly efficacy with minimal toxicity.

Numerous clinical trials and real-world analyses have confirmed the efficacy and safety of blinatumomab, the majority of which were conducted in Western populations (15, 16, 30, 31). Recently, Zhou HS et al. conducted a multicenter, open-label, single-arm trial to evaluate the efficacy and safety of blinatumomab for the first time in Chinese patients with R/R B-ALL (32). The study reported a CR/CRh rate of 47.8%, a median OS of 9.2 months, and a median RFS of 4.3 months after blinatumomab treatment (32). Additionally, Zhou HF et al. performed a retrospective analysis of the treatment patterns, effectiveness, and safety of blinatumomab in Chinese patients with B-ALL in a real-word setting (33). In this study, newly-diagnosed B-ALL patients achieved a CR/CRi rate of 100% and a MRD negativity rate of 87.2%, while R/R patients who received blinatumomab re-induction achieved a CR/CRi rate of 50.0% and a MRD negativity rate of 64.2% (33). The 1-year event-free survival (EFS) rate was 90.8% for newly-diagnosed patients and 55.1% for R/R patients (33). In our study, the CR rate of 80.0% was higher than previously reported, while the MRD response rate of 63.2% that was consistent with earlier studies. This higher CR rate in our study could be explained by the small sample size that resulted in a wider confidence interval (95%CI: 51.9-95.7) and the low proportion of patients with high tumor burden. Previous trials have shown that patients with high tumor burden (≥ 50% bone marrow blast) had significantly lower CR/CRh rates compared with those with < 50% blast (14, 32). In these two trials that reported CR/CRh/CRi rates of 40-50%, over 75% of patients had ≥ 50% blast (32, 33). In contrast, only 36.8% of patients in our study had high tumor burden. Similar to our study, Shi et al. reported a CR rate of 75% in 8 patients with R/R B-ALL, of which only 4 patients had high tumor burden (34). However, our findings need to be confirmed in future studies with a larger sample size. Nonetheless, these results suggest that blinatumomab is highly effective in inducing CR and eradicating MRD in Chinese patients with R/R B-ALL.

Despite achieving a high hematological CR rate following intensive induction/consolidation chemotherapy, approximately 30% to 50% of adult and 10% to 20% of pediatric patients remain MRD-positive (35, 36). Persistent or relapsed MRD is a marker of resistance to standard chemotherapy and represents a key risk factor for relapse in ALL (37). In adult patients, the 5-year hematological relapse rate for MRD-positive individuals ranges from 56% to 100%, compared to 18% to 30% in MRD-negative patients (38). A meta-analysis of 39 studies involving 13,637 patients concluded that both pediatric and adult patients who achieved MRD negativity had significantly improved OS and EFS compared to those who did not (39). In the RIALTO trial, 52% of pediatric patients with R/R B-ALL achieved CR with MRD response within two cycles of blinatumomab, resulting in significantly prolonged OS compared to MRD non-responders (40). However, no difference in RFS between MRD responders and non-responders was observed in this study (40). Conversely, the phase I/II MT-103–205 study reported a longer median RFS in R/R patients who were MRD-negative compared to those who were MRD-positive following blinatumomab (7.0 months *vs.* 1.9 months) (19). A multi-center, real-world study also observed that MRD responders had superior OS and EFS compared to non-responders (31). In our study, R/R B-ALL patients who achieved MRD negativity had significantly improved OS compared to those who did not. These findings support the survival benefit of using blinatumomab to eradicate MRD in R/R B-ALL patients.

In this study, we further analyzed the survival benefit of allo-HSCT after blinatumomab. Among 16 patients who had CR after blinatumomab, no significant OS and RFS differences were found between patients who received HSCT and those who did not. However, several previous studies have reported OS benefit of allo-HSCT after blinatumomab. In RIALTO trial, there was a trend toward improved OS for patients who received allo-HSCT after blinatumomab as compared with those who did not (41). Another trial also reported longer OS in patients who received allo-HSCT compared with those who did not (42). This discrepancy may be caused by the inclusion of patients who did not response to blinatumomab treatment into the survival analysis. Patients who are not in CR after blinatumomab definitely have shorter OS and are less likely to proceed to HSCT. Therefore, the OS benefit of HSCT after blinatumomab may be overestimated due to the inclusion of more patients without CR into non-HSCT group in these trials (41, 42). This explanation is supported by the other two trials (30, 43). Jabbour et al. reported that post-blinatumomab HSCT conferred a survival advantage compared with no HSCT in the total study population (43). However, this advantage disappeared among patients who achieved CR/CRh/CRi, suggesting that the survival may be driven by response to blinatumomab but not HSCT (43). Similarly, Beneduce et al. found marginally significant OS benefit of allo-HSCT in the analysis of the overall study patients, whereas not OS benefit of allo-HSCT among patients who achieved CR after blinatumomab was observed (30). These findings suggest that blinatumomab may provide an alternative to HSCT for potential cure in some patients (43). However, these results may be influenced by confounding factors, such as small sample size, limited follow-up duration, transplant-related mortality, and donor matching. Notably, in our study, two patients who achieved CR and MRD negativity and subsequently proceeded to allo-HSCT experienced transplant-related death. Therefore, the survival benefit of post-blinatumomab HSCT, particularly in patients with CR after blinatumomab, needs further confirmation in future studies.

## Conclusion

Our study demonstrates that blinatumomab is effective and well-tolerated in Chinese patients with R/R B-ALL, inducing high rates of CR and MRD negativity and allowing more patients to proceed to allo-HSCT. However, due to the retrospective nature, small sample size, and short follow-up period of this study, further validation in well-designed, prospective, randomized trials with larger sample sizes is required.

## Data Availability

The raw data supporting the conclusions of this article will be made available by the authors, without undue reservation.
